# Study protocol for type II hybrid implementation-effectiveness trial of strategies for depression care task-sharing in community health stations in Vietnam: DEP Project

**DOI:** 10.1186/s12889-023-16312-4

**Published:** 2023-07-28

**Authors:** Victoria Khanh Ngo, Thinh Toan Vu, Quan Anh Vu, Ryan McBain, Gary Yu, Ngoc Bao Nguyen, Hien Mai Thi Nguyen, Hien Thi Ho, Minh Van Hoang

**Affiliations:** 1 Center for Innovation in Mental Health, Graduate School of Public Health & Health Policy, The City University of New York, New York, NY US; 2 Department of Community Health & Social Sciences, Graduate School of Public Health & Health Policy, The City University of New York, New York, NY US; 3 RAND Corporation, Santa Monica, CA US; 4 Columbia University, New York, US; 5 Hanoi University of Public Health, Hanoi, Vietnam; 6 Vietnam Psychotherapy Association, Hanoi, Vietnam

**Keywords:** Implementation science, Depression care program, Quality implementation, Multi-component collaborative care for depression, Usual implementation, Enhanced supervision, Collaborative learning, Community health stations, Primary care, Vietnam

## Abstract

**Background:**

It is not clear what the most effective implementation strategies are for supporting the enactment and sustainment of depression care services in primary care settings. This type-II Hybrid Implementation-Effectiveness study will compare the effectiveness of three system-level strategies for implementing depression care programs at 36 community health stations (CHSs) across 2 provinces in Vietnam.

**Methods:**

In this cluster-randomized controlled trial, CHSs will be randomly assigned to one of three implementation conditions: (1) Usual Implementation (UI), which consists of training workshops and toolkits; (2) Enhanced Supervision (ES), which includes UI combined with bi-weekly/monthly supervision; and (3) Community-Engaged Learning Collaborative (CELC), which includes all components of ES, combined with bi-monthly province-wide learning collaborative meetings, during which cross-site learning and continuous quality improvement (QI) strategies are implemented to achieve better implementation outcomes. The primary outcome will be measured based on the RE-AIM framework (Reach, Effectiveness, Adoption, Implementation quality, and Maintenance) using indicators on implementation, provider, and client factors. The secondary outcome examines factors associated with barriers and facilitators of quality implementation, while the tertiary outcome evaluates the incremental cost-effectiveness ratio of services provided in the ES and CELC conditions, relative to UI condition for depression care. A total of 1,296 clients receiving depression care at CHSs will be surveyed at baseline and 6-month follow-up to assess mental health and psychosocial outcomes (e.g., depression and anxiety severity, health function, quality of life). Additionally, 180 CHS staff and 180 non-CHS staff will complete pre- and post-training evaluation and surveys at baseline, 6, 12, and 24 months.

**Discussion:**

We hypothesize that the additional implementation supports will make mental health service implementation superior in the ES and CELC arms compared to the UI arm. The findings of this project could identify effective implementation models and assess the added value of specific QI strategies for implementing depression care in primary care settings in Vietnam, with implications and recommendations for other low- and middle-income settings. More importantly, this study will provide evidence for key stakeholders and policymakers to consider policies that disseminate, scale up, and advance quality mental health care in Vietnam.

**Trial registration:**

NCT04491045 on Clinicaltrials.gov. Registered July 29, 2020.

**Supplementary Information:**

The online version contains supplementary material available at 10.1186/s12889-023-16312-4.

## Background

Depression is one of the largest health-related burdens and a high priority for the Global Mental Health Grand Challenge Initiative, the National Institute of Mental Health, the World Health Organization, the World Bank, and the United Nations [1–3]. Its early onset and chronic nature make it particularly injurious to an otherwise productive segment of society [4], by diminishing work capacity [5, 6], increasing mortality from comorbid health conditions and suicide [7], and impairing quality of life and relationships [8]. Globally, effective treatments for depression have been developed and well tested, yet the treatment gap remains unacceptably large: over 75% of those needing services do not receive care, and this gap is even larger in low- and middle-income countries (LMICs) [9–11]. Remarkably, the burden of depression—if unaddressed—will result in crippling economic costs, with more than 12 billion days of lost productivity every year at an estimated cost of US$925 billion [12]. A global return-on-investment analysis for 36 countries between 2016 and 2030 projected that investing a cost of US$91 billion to scale up depression care could lead to economic returns of US$230 billion and health returns of US$250 billion, with an estimated benefit-to-cost ratio of 5.3 (range 4.2–5.7) [12]. This five-fold return on depression care makes a compelling case for global investment in depression care, which resulted in the recent integration of mental health (MH) into the United Nations development goals [13]. Thus, understanding the best strategies for implementing quality evidence-based depression care, particularly in LMICs, is urgently needed.

Collaborative care is a recommended health system quality improvement (QI) intervention to integrate depression care into primary care settings using a team-based approach [14]. In a collaborative care model, a MH specialist supports non-specialists, typically primary care providers, to provide routine screening, health education, evidence-based treatment, and follow-up support. The evidence base for collaborative care for depression in primary care is strong, with over 90 trials showing its effectiveness (including in LMICs) and association with improvements in symptom severity and disease remission [15–18]. For example, we developed the Multicomponent Collaborative Care for Depression (MCCD) program in Vietnam to task-shift depression care to primary care from 2017 to 2019. This randomized controlled trial (RCT) (n = 473) comparing the more comprehensive MCCD with guideline antidepressant medication found significant and large treatment effects for MCCD across all time points and outcomes [19, 20]. Despite strong evidence for collaborative care for depression globally, depression care is not widely implemented in accordance with evidence-based practices. Additionally, MH care has not kept pace with improvements in physical health care, and the quality of MH care may be worsening rather than improving, even in the U.S [21]. Therefore, it is critical to focus on identifying as well as enhancing factors that support scale-up efforts and implementation of such effective MH collaborative care programs.

Globally, there remains limited research on identifying the best training models for task-sharing evidence-based interventions (EBIs) in primary care settings in LMICs. For instance, our first MCCD study in Vietnam primarily focused on evaluating the effectiveness of the enhanced supervision model and failed to examine the effect of the training method on implementation outcomes or to document the intensity or fidelity of enhanced supervision [20]. Furthermore, EBIs using enhanced supervision components have not been compared to other implementation strategies in MH task-sharing, which has been cited as a major contributor to the research-to-practice gap [22, 23]. Establishing efficacious and feasible training models given local resource constraints is critical to effectively implement and sustain EBIs. More importantly, the scarcity of MH expertise and human resources is a reality in LMICs [24, 25], necessitating the need to rely on primary care and lay health workers to provide MH care [26]. However, little is known about what strategies would lead to successful implementation and sustainment of EBIs, particularly in the context of task-shifting in resource-limited settings [27] for both LMICs and high-income countries [28, 29]. Thus, an RCT comparing implementation strategies for task-shifting depression care both directly addresses the treatment gap in LMICs and further advances implementation science more broadly. With a weak MH system governance structure and associated policies, legislation, and effective action plan [30], Vietnam offers an excellent opportunity to empirically test implementation approaches and examine contextual and organizational factors associated with implementing and sustaining EBIs for LMICs.

Previous studies showed that the community-based participatory approach—a widely recommended strategy for addressing health disparities [31, 32]—can increase the program penetration and reach of EBIs in low-resource community settings. Community-partnered processes can engage and activate community members to contribute to healthcare improvement efforts, empower them to take leadership, and even hold public health services accountable to quality improvement plans [33–36]. In depression care, the Community Partners in Care study tested the added value of Community Engagement and Planning (CEP) for implementing collaborative care for depression to the standard approach of providing resources and training to individual agencies [35]. Compared to the standard approach, CEP resulted in significant improvements in the care system, including reduced hospitalizations, homelessness, and increased access to depression care in non-traditional settings [37–39]. Hence, our Community-Engaged Learning Collaborative (CELC) will combine Quality Improvement Collaboratives (QIC) with CEP, adapting core elements most critical for LMICs. This study aimed to address critical implementation research gaps by addressing three key issues: (1) comparing the effectiveness of three implementation strategies: usual implementation (UI), enhanced supervision (ES), and community-engaged learning collaborative (CELC); (2) evaluating organizational and provider factors associated with adoption and implementation quality; and (3) identifying the incremental cost-effectiveness of the ES and CELC arms relative to UI arm for depression care in primary care settings.

## Methods

### Study design

**Fig. 1:**
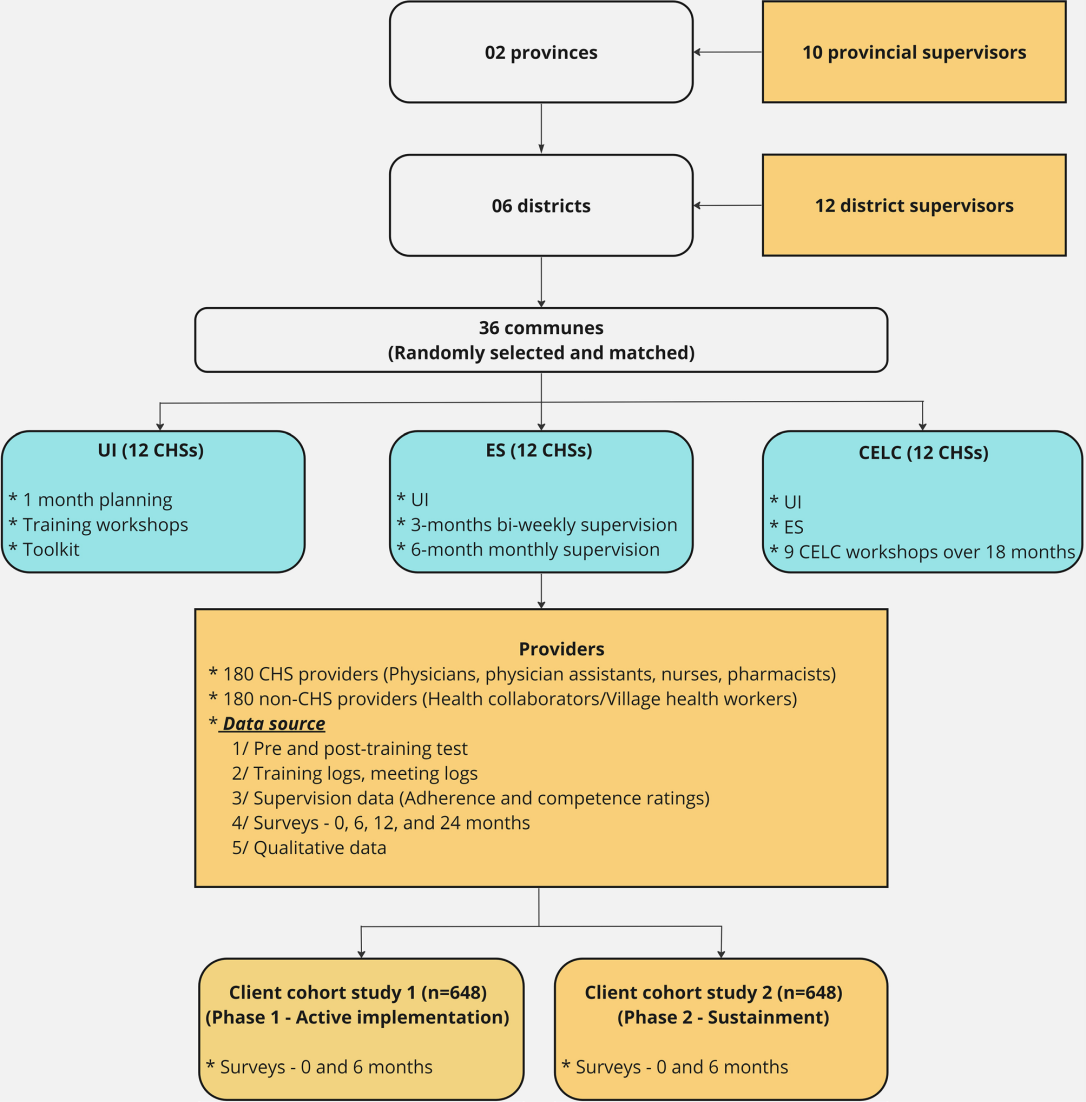
Study design diagram *UI: Usual implementation; ES: Enhanced supervision; CELC: Community-engaged learning collaborative; CHSs: Community Health Stations*

This Type-II Hybrid Implementation-Effectiveness study [40] will primarily test implementation strategies on provider adoption and implementation quality. We will use a cluster RCT design to test the effectiveness of the implementation models on provider- and client-level outcomes with a mixed-methods approach. Specifically, this three-arm trial compares the three multi-component strategies for implementing depression care guidelines: (1) UI arm which includes basic depression care capacity workshops, limited technical assistance, and toolkits, 2) ES arm which includes all components of UI and additional structured clinical supervision from provincial and district supervisors, and 3) CELC arm which includes all components of ES, combined with the activation of a community-wide network of providers and stakeholders who are implementing continuous QI strategies (Fig. 1). The primary outcome is to compare the fidelity of MCCD on three implementation models using the RE-AIM framework [41], which will be assessed based on implementation outcomes (**R**each, **A**doption, **I**mplementation quality, and **M**aintenance) and provider and client-related outcomes (**E**ffectiveness) (Table 1). The secondary outcome is to assess factors associated with barriers and facilitators of quality implementation, which may serve as mechanisms for implementing additional supports for the ES and CELC arms. The tertiary outcome is to evaluate the incremental cost-effectiveness which quantifies the cost savings to policymakers when integrating various strategies for task-shifting depression care into primary care settings.

**Table 1:** RE-AIM Indicators, Description, and Data Sources

Indicator	Description	Source
**Training Participation**		
Workshops	Provider participation in workshops	Workshop Attendance Log
Supervision	Provider participation in Supervision	Supervision Log
Learning Collaborative (LC)	Provider participation in LC meetings	LC Attendance Log
**Reach**		
Screening	Total # of people who were screened	Clinical Form
Individual Therapy - Behavioral Activation (BA)	Total # and percentage of BA treatment acceptance	Clinical Form
Medication	Total # and percentage of medication prescription	Clinical Form
**Adoption**	Provider self-reported depression care practices at 6, 12, 24, and 36-month follow-up	Provider Survey
**Implementation Quality**		
Adherence	Degree of fidelity to the content of the BA sessions	Supervision Ratings
Quality	General quality implementing the BA sessions	Supervision Ratings
Depression Care Competence	Depression care competence rated by supervisors	Provider Competency Assessment
Treatment Completion	# and % of clients with 4 or more sessions	Session Progress Note
Treatment Dropout	# and % of clients with 1 or less sessions	Session Progress Note
**Effectiveness**	Pre- and post-treatment differences; PHQ-9 Survey data	PHQ-9 Treatment Data and Survey Data
Client Improvement	50% reduction in PHQ-9 score	PHQ-9 – Session Scores (first and last)
Client Remission	Last PHQ-9 < 5	PHQ-9 – Session Score (last)
**Maintenance**	Independent implementation is considered an early sign of maintenance or penetration	Active Implementation Support; Transitional Support

### Study site eligibility and recruitment

We are recruiting 36 community health stations (CHSs) from two provinces (Bac Giang and Phu Tho) that are located within a one-to two-hour drive from Hanoi capital. CHSs from each province will be randomly assigned into one of three intervention conditions with two implementation support phases: Phase 1—Active Implementation Support, and Phase 2—Sustainable Support (e.g., after supervision is withdrawn from the international training team and transitioned to the local supervision team). The recruitment process involves (1) identifying three potential districts per province based on recommendations from the provincial health department and psychiatric hospital, (2) conducting engagement meetings with three district-level health leaders to introduce the project and expectations, (3) conducting engagement meetings with CHS leaders to introduce the program, and (4) inviting interested CHS administrators to submit a form indicating interest in participating, as well as willingness to be randomized, and to complete a brief CHS assessment survey.

CHS selection criteria include having at least one physician/physician assistant, ≥ 5 CHS staff, ≥ 5 non-CHS staff including health collaborators/Village health workers (VHWs), serving a population of at least 5,000, and of which, ≥ 200 clients visit CHS monthly. We will score CHSs based on two main categories: (1) *site characteristics* (e.g., distance to district health centers and provincial psychiatric hospital, staff-to-client ratio, number of clients per month); and (2) *MH indicators* (e.g., implementing any MH programs, management and treatment for schizophrenia and epilepsy clients, accessibility to psychotropic medications). Given the total summary scores, CHSs will be classified as high- and low-performing sites. We will use Statistical Analysis Software (SAS) version 9.4 to randomly assign CHSs within a province into one of three conditions and the randomization process occurs within matched sites within district level, resulting in 6 UI, 6 ES, and 6 CELC. Each intervention arm will include 3 high performers and 3 low performers. Due to cost and staffing, we are implementing intervention at 18 CHSs in Bac Giang in the first year (2022) and will be continuing at 18 CHSs in Phu Tho in February, 2023.

### Description of intervention conditions

#### Conceptual framework

**Fig. 2:**
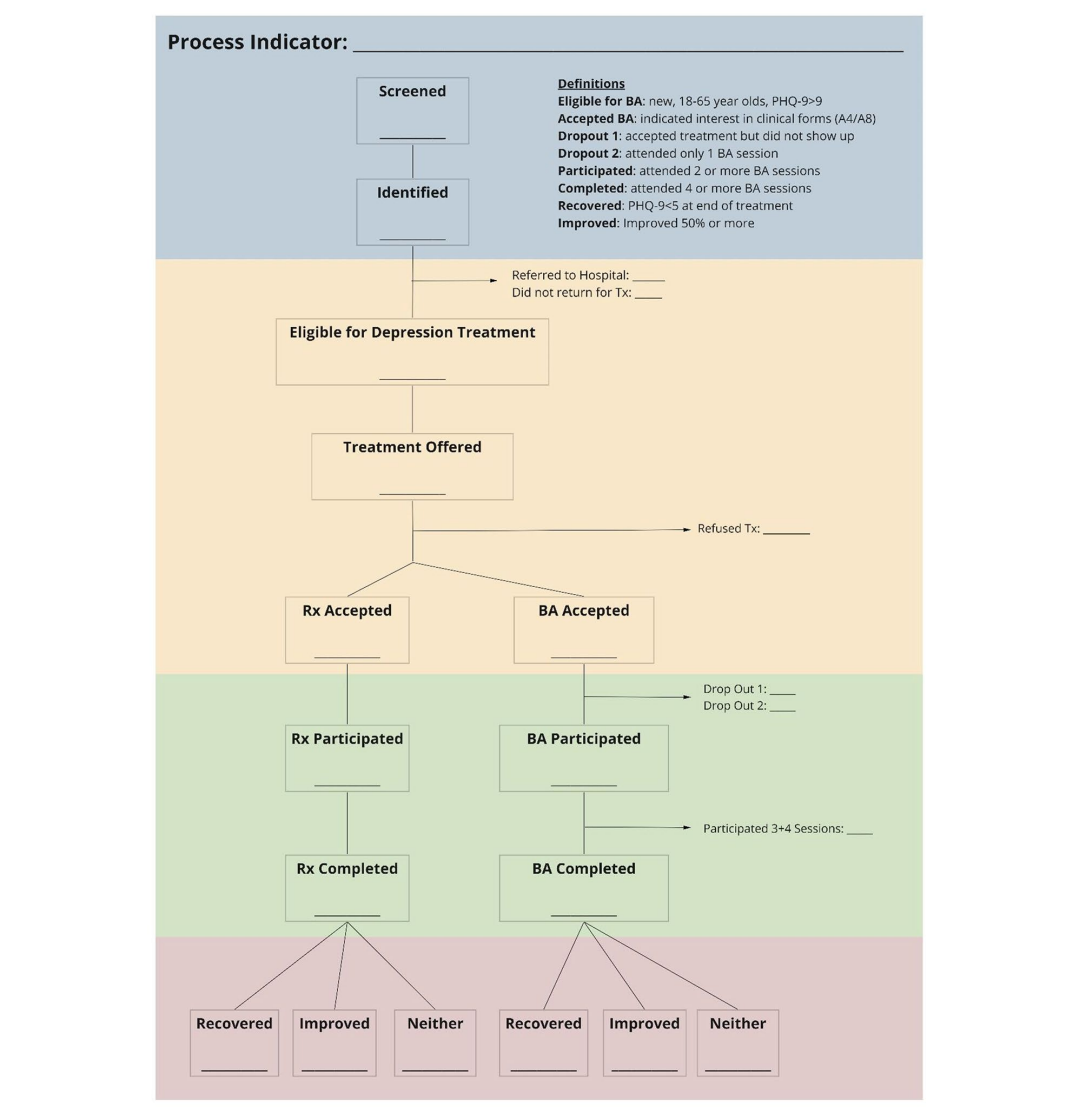
Process indicator diagram *BA: Behavioral activation; Rx: Treatment; PHQ-9: Patient Health Questionnaire with 9 items*

Our intervention draws on the Multicomponent Collaborative Care for Depression model [19, 20] that includes the following clinical processes: (a) Step 1: Community engagement and MH promotion to educate the community about depression, the importance of quality MH services in the community, and details about the MCCD program that is currently available at CHSs (b) Step 2: Screening and assessing at-risk groups in the community for signs of clinical depression and other comorbid MH conditions (e.g., suicidality, mania, psychosis, and alcohol use); (c) Step 3: Psycho-educating new incoming clients about their own depressive symptoms to raise awareness, promote help-seeking behaviors, reduce stigma, and commit to depression care; (d) Step 4: Providing Behavioral Activation (BA), a six-sessions therapeutic intervention adapted from the Building Recovery by Improving Goals, Habits, and Thoughts programs [42, 43] used in We Care [44] and Community Partner in Care [35], with its behavioral treatment components based on Beck’s Cognitive Behavioral Therapeutic model [45]. The learning content in BA covers key topics for behavior-emotion management treatment, such as the connection between activities and mood, increasing healthy and pleasurable activities, overcoming obstacles through effective problem solving, and maintaining healthy habits; (e) Step 5: Prescribing guideline antidepressant medication (e.g., amitriptyline) via certified psychiatrists working at the provincial psychiatric hospital; and (f) Step 6: Community follow-up, which provides ongoing support when needed. The process indicators for the stepped-care model for depression are presented in Fig. 2.

According to the stepped-care treatment model, mild to moderately depressed clients (with Patient Health Questionnaire (PHQ-9) scores between 10 and 20) are offered BA as first-hand treatment, due to its non-invasive nature. Clients who do not respond to BA treatment after four weeks of therapy or who, for extenuating reasons, exhibit worsening depressive symptoms (PHQ-9 > 20) will be offered insurance-paid, guideline antidepressant medication (e.g., Amitriptyline) following standard medication prescription algorithm set by provincial psychiatric hospitals. At the top step, those with severe and treatment-resistant depression (PHQ-9 > 20 without reduction), developing high risk of suicide or other MH comorbidities will be referred to the provincial psychiatric hospital for further clinical evaluation and treatment. This model allows for more effective shifting of depression care tasks from the provincial-level psychiatric hospital, through the District Health Centers, to the local CHSs so that quality primary MH care services become more integrated into the community and readily available for people in need. The effectiveness of this program has been shown previously in an RCT conducted in Vietnam [20]. However, it is not clear what the added value and effectiveness of additional implementation supports, such as enhanced supervision and community-engaged learning collaboratives may be. Therefore, selected CHS will be randomly assigned into 1 of the 3 implementation arms, each of which will receive different levels of implementation support as described below:

### Arm 1: usual implementation (UI)

#### Staff

All CHS staff will attend a series of hybrid depression care capacity training which includes a total of five weeks of online self-learning modules, four in-person workshops and five online Zoom-based webinars (totaling approximately 50 hours of hybrid training), implementation toolkits (e.g., BA manual, program implementation manual, clinical forms, workflow, clinical flow charts, health education materials), and limited technical support from national-level training team (e.g., initial program setup, observing depression screening and assessment) prior to the official rollout of the depression care program. All CHS staff will be trained to document service delivery using documentation tools, such as the screening log, screening and clinical intake assessment forms, session notes, and other clinical support forms. These forms will serve as the program’s administrative records. Additionally, non-CHS staff will attend a MH education and community outreach training which include two-week online learning on Moodle system and two-day in-person workshop. All non-CHS staff will be trained on promoting and educating MH in the community, screening for depression using the PHQ-4 as well as referring at-risk people and supporting care navigation to ensure successful linkages to CHSs.

#### Toolkits

CHS providers will be given a collection of implementation tools, including an implementation guide that lists out step-by-step how to set up and implement depression care services, a detailed manual on how to provide BA therapy, the primary depression treatment intervention of the project. Clinical and research workflows are placed in visible locations inside the CHSs so that providers can always stay informed about the depression care steps and their new roles and responsibilities as MH professionals. Additionally, implementation forms will be printed out and given to CHS providers to assist them in documenting clinical activities and to collect implementation data for the research study.

### Arm 2: enhanced Supervision (ES)

CHS providers will receive the same training and toolkits as those in the UI group, with an additional nine months of bi-weekly to monthly group supervision support from national, provincial, and district-level supervisors. Providers will receive three-hour biweekly in-person group supervision during the first three months of post-training project implementation. This is followed by monthly group supervision for the remaining 6 months of phase 1—Active Implementation Support. Clinical group supervision/consultation provided by provincial- and district-level health providers has three aims: (1) to support implementation activities and problem-solve barriers to implementation at the CHS; (2) to enhance CHS providers’ skills in providing BA by observing sessions and providing structured feedbacks (e.g., adherence and quality rating scale); (3) to assess CHS providers’ depression care competence every 6 months.

### Arm 3: community-engaged learning collaborative (CELC)

CHS Providers will receive all components of the ES group, plus a new quality improvement (QI) strategy: CELC. The CELC meetings will be based on the community engagement and planning implementation intervention model in Community Partner in Care [35, 38, 46] and the CELC from the Institute for Healthcare Improvement (IHI) Chronic Care Model [47]. The IHI model includes key components like forming a planning group that decides on a mutual set of target objectives, identifies specific areas for change, prepares pre-work with participants, implements in-person learning sessions. During CELC meetings, CHS staff and their supervisors learn about QI approaches and strategies on how to provide effective ongoing support to clients (e.g., phone calls, visits, messaging via apps, social media). Between each learning session, the CELC groups engage in Plan-Do-Study-Act (PDSA) cycles in which they brainstorm and decide on brief CHS-level interventions aiming to improve certain clinical processes at the CHS or meeting certain treatment benchmarks and assess their impact afterward [48]. Because of the resource intensity of the IHI model, we will adapt and simplify the approach in partnership with the community-academic-policy council to develop a learning collaborative (LC) that is feasible and culturally appropriate for the Vietnam health and social system. For example, QI learning collaboratives tend to last between 1 and 2 years [49], but we will only have nine meetings that are strategically placed to maximize implementation support.

After the initial LC meeting, the CELC arm will meet every other month to learn and improve their clinical capacity, review their implementation progress, develop strategies to improve implementation in the community (e.g., raise awareness about depression, more effective BA treatment) and create linkages to stakeholder community organizations (e.g., Woman’s Union, People’s Committee), with the larger goal of facilitating the maintenance and scaling up of quality, cost-effective depression care services in primary care settings throughout the province. In an effort to reduce burden on providers, we will not hold LC meetings during the initial intensive supervision period (e.g., Months 1 to 4 post training) so that providers can focus on skill-building and implementing MCCD in their CHSs. We will begin the LC meeting series alongside 6 months of monthly supervision supports to the CHS. Each CHS in the CELC arm will develop and follow up with its direct provincial/district supervisors about their project implementation and sustainability plan.

### Evaluation and analysis plan

Mixed methods, including in-depth interviews and surveys, will be collected using clinical forms, completed by clients and providers. The evaluation and analysis plan are organized according to the following domains by aims: RE-AIM outcomes (Fidelity to MCCD), barriers and facilitators of implementation, and cost-effectiveness.

### Outcome evaluation and analysis for AIM 1 (Fidelity to MCCD)

#### Power analysis

We used the pwr package in R software to determine the sample size and estimate power to detect a small eta-squared effect size of 0.025 with an expected intra-class correlation coefficient (ICC) of 0.2. Our primary outcome analyses focus on the 12-month timepoint, we will enroll 10 providers per site resulting a total of 360 providers (180 CHS staff and 180 non-CHS staff) across 36 sites in order to achieve 80% power to detect the small effect size. We also enroll 18 clients per site per phase which results in a total of 648 clients over two phases. With client outcome, power calculations assume near-normal outcomes with a two-sided Type I error of 0.05, this design has 80% power to detect small effects (small Cohen’s d effect size of 0.14 for a 3 × 3 contingency table chi-square test, given 4 degrees of freedom [(3 intervention groups {UI, UI + ES, UI + ES + CELC} − 1)*(3 outcome levels of PHQ-9 score change from baseline to 6 months {positive change, no change, negative change} − 1)]).

#### RE-AIM outcomes

According to the RE-AIM framework, for an intervention to have an impact, it must **Reach** the population in need of the service, demonstrate **Effectiveness**, be **Adopted** by the system and providers, be **Implemented** with quality, and be **Maintained** or sustained over time. The indicators’ description as well as data sources were presented in detail in Table 1. A cascade of MH care will be built based on these indicators (Fig. 2). Mean and standard deviation will be presented without the normality violation, otherwise, median and interquartile ranges will be used. Independent ANOVA and/or Kruskal-Wallis test will be applied to assess differences stratified by intervention arms.

#### Provider selection

Based on power considerations, we will recruit 10 providers from each CHS and 360 across the study, including five primary care providers (usually physician assistants or nurses) from the CHS and five (usually VHWs) working with the CHS, totaling 180 CHS providers and 180 non-CHS staff. Providers will be recruited post-enrollment but pre-CHS randomization at each CHS.

We will use a repeated measure design to conduct online self-reported surveys with CHS and non-CHS providers at baseline (before the workshop, during the study enrollment meeting), 6-, 12-, and 24-month follow-up. All follow-up surveys will be distributed and collected by the research coordinator within a one-week period of each data collection time point. The survey will assess the following categories: socio-demographic characteristics (e.g., year of birth, gender, education, number of years working in health system), MH training, depression care attitude scale using the adapted Evidence-Based Practice Attitude Scale (EBPAS) [50], working environment using the Implementation Climate Scale (ICS) [51], CHS head’s leadership using the Implementation Leadership Scale (ILS) [52], depression care activities (e.g., frequency of depression screening and depression treatment offered) and skills, depression stigma [53] and depression knowledge quiz including 20 multiple-choices questions which covers all training topics.

#### Client outcomes

Clients identified with depression as part of routine depression screening at CHS will be recruited to participate in the client outcomes survey study across two implementation support phases. Eligible clients included those who are (1) new to the program, (2) aged 18–65, and (3) screened positive for depression using the PHQ-9 (score > 9). Those with a risk for severe mental illness (psychosis, mania, and substance abuse) will be excluded from the Client Study Cohort and referred to the provincial psychiatric hospital, per the stepped care model mentioned in the intervention description. Based on a depression screening rate of 20% and a research acceptance rate of 75% from our prior work [20], we expect CHS staff to screen 2,200 clients to consent 324 participants across each province for each Client Study Cohort. Across the total sample of 648 clients in each implementation phase. We will request permission from participants to link survey data to client administrative and clinical data.

The effectiveness on the client outcome will be measured based on the client’s improvement (defined as a 50% reduction in PHQ-9 score between the first and last BA session) and client’s remission (defined as the last PHQ-9 score less than 5). We will conduct an intent-to-treat analysis (e.g., those who are lost to follow-up are considered neither improved nor remised) in order to ensure the randomization process. A generalized linear mixed-effects model will be used for binary outcomes with a binominal family distribution and logit link. Exponentiating the fix-effect coefficients for treatment conditions will result in estimates of the odds ratio regarding the effect of treatment condition on depression symptomatology. These models will be adjusted for the clustering effects across multiple levels of hierarchical data structure (e.g., providers, clients) and the UI intervention will be used as a reference group.

In addition to the PHQ-9, our client cohort study will collect additional information at baseline and 6-month surveys, including socio-demographic information (e.g., age, gender, education, marital status, household characteristics), house and general socio-economic status (e.g., house ownership, monthly expense, social standing), employment and business activities (e.g., employment status, regular source of income, personal and family monthly income), health functioning using the World Health Organization Disability Assessment Schedule 2.0 (WHO-DAS 2.0) [54], quality of life (Q-LES-Q-SF) [55], self-efficacy using the adapted Adult AIDS Clinical Trials Group (AACTG) [56], depression stigma [53] and Behavioral Activation for Depression Scale (BADS-SF) [57], social support using the Medical Outcomes Study (MOS) [58], social capital, family environment using the McMaster Family Assessment Device (FAD) [59] and CHS Client Satisfaction Questionnaire (CSQ) [60].

### Outcome evaluation and analysis for AIM 2

The secondary outcome is to assess factors associated with barriers and facilitators of quality implementation. Implementation progress, barriers, and facilitators will be captured using mixed methods, which will combine monthly implementation data with qualitative interviews for high and low-performing sites (high and low adopters) in each province for both phases. Interviewers should review the implementation data table before conducting qualitative interviews to construct the “implementation story” based on the monthly implementation data which is extracted from clinical records/logs and training records. Interviewers will interview all providers, leaders, and supervisors engaged in depression care for each site, but not all questions need to be asked for each provider. We expect to interview approximately 5 participants (2–3 providers, 1 leader, and 1 supervisor) to construct the implementation story, including milestones, timelines, barriers, and facilitators to implementing and sustaining depression care. We also assess their feedback regarding toolkits and materials, training, supervision support, and learning collaboration to identify the strengths and weaknesses of our program. These interviews will be conducted at the end of each phase.

Qualitative data will be transcribed verbatim in Vietnamese and translated into English. This process will be conducted by a bilingual doctoral student in community health and health policy (TTV) who had a master’s degree in Epidemiology to ensure the conceptual equivalence of translated transcriptions. All transcripts and descriptors will be imported to Dedoose [61] for data management and analysis. The grounded theory analysis will be implemented to identify themes during the iterative coding process. Further, two research assistants will independently code all transcripts to ensure interrater reliability. All notes will be added to the analytic memos whenever a new code emerged. The research assistants will consistently revise the codebook and re-group the codes used if necessary.

### Outcome evaluation and analysis for AIM 3

Activity-based costing will be used to assess costs associated with all activities involved in the implementation strategy interventions and depression care services, inclusive of (i) the personnel involved, (ii) the infrastructure and physical space utilized, (iii) equipment, (iv) consumables such as medications and print-outs, and (iv) overhead and other indirect costs such as utilities (Table 2). This will also entail an assessment of client costs, in order to determine the full cost of the program from a societal perspective. Team members will gather cost information from electronic financial systems, payroll, facility ledgers, and price lists to ascertain the cost of various medical equipment, infrastructure, personnel and consumables. Salaries will be quantified to include fringe benefits for relevant personnel involved in the study—including the provincial and district supervisor, CHS and non-CHS staff, and our implementation partners. The cost of medical equipment, which will be obtained from facilities records, will be annualized based on linear depreciation over estimated useful lifespan.

**Table 2:** Identification of Main Costs for Each Group of Participants

Measures of Direct Cost	Collected	Source	Valuation
**LABOR COSTS (time spent on…)**			
Depression care services	2,3,4	Monthly labor worksheet	Wage rates
Community outreach activities	2,3,4	Monthly labor worksheet	Wage rates
Planning activities	1,2,3	Monthly labor worksheet	Wage rates
Training activities	2,3,4	Attendance sheets	Wage rates
Monitoring/supervision activities	2,3,4	Supervisor logs	Wage rates
Learning collaborative activities	1,2,3,4	Attendance sheets	Wage rates
Providing technical assistance	1	Supervisor logs	Wage rates
***SUPPLIES***			
Cost of workshop/training materials including toolkit, outreach materials, etc.	1,2	Hanoi University of Public Health accounting records	List price
Food, copies, stationary, other equipment, medications	1,2,3	Monthly accounting records	Local prices
***MISCELLANEOUS COSTS***			
Travel	1,2,3,4	Weekly logs, travel reimbursement	Mileage rates
Lodging	1,2	Weekly logs	Prices paid
***OVERHEAD (INDIRECT) COSTS***			
Capital costs - Space for meetings (sq. feet) - Equipment (e.g., computers)	1,2,3	Weekly logs Key informant interviews	Rental rates
***CLIENT PARTICIPATION COSTS***			
Client time	5	Key informant interviews	Avg income
Client travel	5	key informant interviews	Local prices
Treatment cost	3,5	Invoices, key informant interviews	Service rates

**Key Code**: 1: Project staff providing TA; 2: Local implementation staff; 3: CHS staff members; 4: non-CHS participants in the community collaborative; 5: Clients

We will estimate the incremental cost-effectiveness ratio (ICER) of ES and CELC relative to UI of depression care, defined as the incremental change in costs relative to the incremental change in effectiveness (e.g., QALYs with ES/CELC minus QALYs with UI alone). Effectiveness will be measured based on (1) adoption rate (measured as proportion of trained providers delivering MCCD components); (2) implementation quality measured by average fidelity score; (3) reach (measured as proportion of eligible clients getting treated); (4) effectiveness (measured by client outcomes by using average PHQ-9 scores, work days missed, and QALYs); and (5) maintenance (measured as adoption, reach, implementation quality, and effectiveness at 24 months).

To examine the ICER for each enhanced implementation strategy, a Markov modeling framework [62] will be utilized to estimate the relative costs and outcomes of clients enrolled in each of the three trial arms, with UI serving as the base case by which to compare alternative implementation frameworks. A Markov chain Monte Carlo approach with 100,000 simulations will also be utilized for sensitivity analyses, in which we systematically examine the extent to which variation in cost and implementation parameters affect estimated ICERs.

### Ethical considerations

This study was approved by the Institutional Review Board at the Graduate School of Public Health and Health Policy, The City University of New York, U.S. and The Hanoi University of Public Health in Vietnam.

### Trial status

As of December 2022, we successfully recruited 18 CHSs and provided training to 5 provincial supervisors, 6 district supervisors, 90 CHS staff and 90 non-CHS staff in Bac Giang through a combination of in-person training and Moodle online learning platform. All providers underwent assessments before and after the training. During the phase 1 of the project, we interviewed 324 clients and completed collecting a 6-month follow-up survey for CHS and non-CHS providers. The recruitment of an additional 18 CHSs in Phu Tho will continue in 2023.

## Discussion

Depression is a prevalent and debilitating MH condition that affects people in nearly every country, including Vietnam, and is one of the largest burdens of health [63]. Although effective treatments exist, many with depression do not receive appropriate care [64]. The treatment gap is significantly expanding, especially during the COVID-19 pandemic when global prevalence of depression and anxiety increased by 27.6% and 25.6%, respectively [65]. Likewise, the Vietnamese population experienced a depression burden during the pandemic with six times higher compared to prior period (14.6% vs. 2.5%, respectively) and if these disorders are not addressed appropriately, the community will suffer from significant long-term mental social and economic consequences [66]. Yet, previous MH interventions have primarily targeted high-risk groups such as hospital nurses [67], people who inject drugs [68], caregivers [69], and cancer clients [70]. There are only two RCTs with small sample sizes conducted in the primary care centers [71, 72]. Hence, in order to significantly close the treatment gap as well as sustain treatment for depression, effective depression care programs with a large sample size need to be piloted and scaled up to a wider network of community health stations that are supported by the local psychiatric hospitals.

In response to the increasing recognition of the need to address the treatment gap for depression, Vietnam developed a national plan on MH disorders for the period 2022–2025. The strategy adopts a community-engaged multi-sectoral approach to promote evidence-informed policies and practices to improve MH care [73]. This national strategy focused on implementing key activities, aligning financial resources for MH human resources and infrastructure, building capacity, developing research, information technology, and multi-sectoral coordination mechanisms to achieve its goals. This study directly addresses a pressing issue not only in Vietnam but also within the costly international health crisis during the COVID-19 pandemic. This study, therefore, will play a key role in finding the most effective and cost-efficient strategy for integrating MH care with primary health care in vulnerable and resourced-limited communities.

Our previous work has developed a task-shifting MH care model called the MCCD program, which was proven effective according to an RCT conducted in Da Nang and Khanh Hoa provinces [20]. The model provides psychotherapy for a 2–3 month period and can be delivered by CHS nurses, physicians, and other community health workers who are supervised by local psychiatrists. The study findings suggest that collaborative care for depression can be task-shifted to primary health and community health providers with minimal support from MH specialists. However, the optimal implementation model for supporting depression care in primary care settings, the added value and effectiveness of adding additional implementation supports such as enhanced supervision and community-engaged learning collaboratives, and factors influencing adoption, implementation quality, and sustainability when scaling depression care are not yet clear. So, this study will address the gap by: (1) enhancing intersectoral collaboration and community engagement, (2) increasing capacity to detect and treat MH conditions, particularly depression, in primary care settings, and (3) strengthening information systems, evidence, and research on MH in low-resource settings.

There are several limitations to our study. COVID-19 posed significant challenges to successful implementation of this protocol. Firstly, the COVID-19 pandemic began during the study, causing the transition of in-person training to hybrid training to comply with social distancing policies and travel limitations. The U.S. study staff were unable to support on-site providers in terms of training and practicing key skills in person. Although, CHS providers were able to practice BA in person with clients—the main psychoeducation method in this study, they did not receive direct supervision. This barrier might have a small impact on providers’ skills since their sessions were recorded and provided with feedback during consultation calls. Therefore, project staff along with provincial and district supervisors could help them identify strengths and weaknesses for psychotherapy practices. Another limitation was the limited availability of appropriate staffing due to the deployment of healthcare staff to national COVID-19 testing and vaccination programs in various provinces. However, after training was completed virtually and in-person, the intervention ran smoothly and did not appear to impact implementation of the program. Lastly, our intervention is conducted in two mountainous and rural areas, which limit the ability to compare the effectiveness of depression care program in rural and urban areas. However, the Vietnam healthcare system follows a hierarchal structure, and findings from this intervention—will provide decision-makers with evidence bases about what could be achieved if they enacted strategic adjustments in their approach to health service delivery—could be applicable to other provinces over the country. Again, despite these limitations, the findings of this project hold high implications by identifying best practices for implementing depression care in primary care settings in Vietnam and other low- and middle-income countries. More importantly, this study will provide evidence bases for key stakeholders and policymakers to make decision towards scale-up as well as dissemination of MH care guidelines.

## Electronic supplementary material

Below is the link to the electronic supplementary material.

Supplementary Material 1

## Data Availability

Data collection for this study is ongoing, so no data and materials are currently available.

## Abbreviations

BA: Behavioral Activation
CELC: Community-Engaged Learning Collaborative
CEP: Community Engagement and Planning
CHSs: Community Health Stations
EBIs: Evidence-based Interventions
ES: Enhanced Supervision
ICER: Incremental Cost-Effectiveness Ratio
LMICs: Low- and Middle-Income Countries
MH: Mental Health
MCCD: Multicomponent Collaborative Care for Depression
UI: Usual Implementation
PHQ: Patient Health Questionnaire
QIC: Quality Improvement Collaboratives
VHWs: Village Health Workers
